# Structural Equation Modeling (SEM) Analysis of Sequence Variation and Green Plant Regeneration via Anther Culture in Barley

**DOI:** 10.3390/cells10102774

**Published:** 2021-10-16

**Authors:** Piotr Tomasz Bednarek, Renata Orłowska, Dariusz Rafał Mańkowski, Sylwia Oleszczuk, Jacek Zebrowski

**Affiliations:** 1 Department of Plant Physiology and Biochemistry, Plant Breeding and Acclimatization Institute—National Research Institute, 05-870 Błonie, Poland; r.orlowska@ihar.edu.pl; 2 Department of Seed Science and Technology, Plant Breeding and Acclimatization Institute—National Research Institute, 05-870 Błonie, Poland; d.mankowski@ihar.edu.pl; 3 Department of Plant Biotechnology and Cytogenetics, Plant Breeding and Acclimatization Institute—National Research Institute, 05-870 Błonie, Poland; s.oleszczuk@ihar.edu.pl; 4 Institute of Biology and Biotechnology, University of Rzeszow, 35-959 Rzeszow, Poland; jaze28@interia.pl

**Keywords:** androgenesis, barley, β-glucans, copper ions, DNA methylation, DNA sequence, plant regeneration, silver ions

## Abstract

The process of anther culture involves numerous abiotic stresses required for cellular reprogramming, microspore developmental switch, and plant regeneration. These stresses affect DNA methylation patterns, sequence variation, and the number of green plants regenerated. Recently, in barley (*Hordeum vulgare* L.), mediation analysis linked DNA methylation changes, copper (Cu^2+^) and silver (Ag^+^) ion concentrations, sequence variation, β-glucans, green plants, and duration of anther culture (Time). Although several models were used to explain particular aspects of the relationships between these factors, a generalized complex model employing all these types of data was not established. In this study, we combined the previously described partial models into a single complex model using the structural equation modeling approach. Based on the evaluated model, we demonstrated that stress conditions (such as starvation and darkness) influence β-glucans employed by cells for glycolysis and the tricarboxylic acid cycle. Additionally, Cu^2+^ and Ag^+^ ions affect DNA methylation and induce sequence variation. Moreover, these ions link DNA methylation with green plants. The structural equation model also showed the role of time in relationships between parameters included in the model and influencing plant regeneration via anther culture. Utilization of structural equation modeling may have both scientific and practical implications, as it demonstrates links between biological phenomena (e.g., culture-induced variation, green plant regeneration and biochemical pathways), and provides opportunities for regulating these phenomena for particular biotechnological purposes.

## 1. Introduction

Anther culture involves cold treatment of spikes [1], dark incubation of tissue culture [2], addition of chemicals [3], and many other stress-inducing steps [4] that affect plant regeneration. Cold treatment is necessary for the developmental switch of microspores from the gametophytic to embryogenic fate [5], a process that requires DNA demethylation, followed by de novo methylation [6]. DNA demethylation as, well as de novo methylation, may alter the DNA methylation patterns [7,8,9,10], which could lead to DNA mutations [11] and mobile element activation [12,13]. During reprogramming, an increase in cell death and oxidative stress is observed [14]. The equilibrium between reactive oxygen species (ROS)-scavenging and ROS-producing mechanisms governs the level of damage and oxidative stress in the cell [15,16]. Low temperature may also alter endogenous ethylene (ET) levels, enhancing tolerance to higher ET concentrations [17]. Dark incubation of tissue culture in the presence of mannitol leads to carbon starvation [18], forcing cells to utilize all available carbon resources [19,20]. Because photosynthesis cannot be accomplished in the dark, cells attempt to utilize *β*-glucans located between the cell wall and cell membrane, the so-called subintinal layer, in many kinds of cereals. In barley (*Hordeum vulgare* L.), β-glucans synthase are encoded by the *CELLULOSE SYNTHASE*-*LIKE* (*CSL*) gene family [21]. In cells that undergo fate transition, the so-called subintinal layer usually contains callose (β-1,3-glucan) [22]. Callose protects the cell from disruption due to osmotic stress and can be easily utilized by the cell for glycolysis [23,24]. Moreover, glucans also act as antioxidants, thus lowering oxidative stress and affecting the mitochondrial respiratory chain in humans [25]. Moreover, in cultured tobacco cells NADPH-dependent enzymes are involved in glucan-elicited resistance responses, and the inhibition and enhancement of ROS production using NADP^+^ and NADPH, respectively [26]. Unfortunately, it is unclear whether *β*-glucans lower oxidative stress in plants. However, during the shortage of glucose, glycolysis is disturbed, which affects the tricarboxylic acid cycle (TCA) [27]. Tissue culture is also affected by the presence of ingredients, such as copper (Cu^2+^) and silver (Ag^+^) ions. Cu^2+^ ions participate in the mitochondrial electron transport chain [28,29], photosynthesis and respiration [30,31], ET detection [32], cell wall metabolism [28], and oxidative stress [33]. Cu^2+^ ions also contribute to hydroxyl radical formation [29] and affect the Krebs cycle at high concentrations [34]. Moreover, Cu^2+^ ions act as a cofactor for the binding of ET to the ethylene receptor 1 (ETR1) protein [32], and participate in a broad range of biochemical pathways linked with DNA methylation [35]. It was also reported that the higher level of copper concentration in the medium was beneficial for the frequency of green plants regeneration in various in vitro cultures [36,37]. Increasing efficiency in the regeneration of green plants is significant, especially in the androgenesis process, where albinism is one of the major problems reducing the number of obtained plants [38]. Albino regenerants (off type) have an impaired chlorophyll production mechanism [39] and are thus unable to carry out photosynthesis (about albinism see more in [40]). Because copper is involved in chlorophyll biosynthesis [41], plastid division and the transformation of amyloplasts into proplastids, therefore, can affect the number of albino plants [42]. In addition to Cu^2+^ ions, Ag^+^ ions also affect tissue culture; for example, Ag^+^ ions increase the number of green plants derived via androgenesis [43]. Cu^2+^ and Ag^+^ ions are of comparable sizes. Thus, Ag^+^ ions may replace Cu^2+^ ions [44], for example, in the mitochondrial complex IV [45]. Both ions form complexes with ET [46,47]. Additionally, Ag^+^ ions influence ethylene action, thus inhibiting its receptors [46].

Although abiotic stresses and ingredients used in tissue culture media during anther culture are required for the regeneration of green plants, these stresses alter DNA methylation patterns and induce DNA sequence variation (SV). Recently, we determined the quantitative characteristics of barley regenerants derived by anther culture using methylation-sensitive amplified fragment length polymorphism (metAFLP) molecular markers [9], and mediation analysis of these quantitative characteristics demonstrated that Cu^2+^ and Ag^+^ ion concentrations in the medium and culture duration affect CG demethylation, leading to DNA mutations [48]. Additionally, mediation analysis of the spectral features of barley regenerants obtained using attenuated total reflection Fourier transform infrared (ATR-FTIR) spectroscopy, which is sensitive to the presence of molecular structures and their molecular environment [49], revealed that β-glucans, and S-adenosyl-L-methionine (SAM) participate in DNA methylation, resulting in SV [50]. The ATR-FTIR spectroscopy method has been successfully used for examination of biological material, including plant tissues [51,52,53]. Finally, using diversity arrays technology sequencing methylation analysis (DArTseqMet) [54] markers and moderation analysis [55], we confirmed that Cu^2+^ and Ag^+^ ions are involved in green plant regeneration in barley [56]. However, relationships between β-glucans, DNA methylation patterns, Cu^2+^ and Ag^+^ ions, SV, green plant (GP) regeneration, and anther culture duration were not evaluated.

We hypothesized that abiotic stresses acting on barley microspores are somehow sensed by the inner callose layer comprising β-glucans. Cells utilize β-glucans as a carbon source under in vitro culture conditions, thus affecting glycolysis and TCA. The presence of Cu^2+^ and Ag^+^ ions in the medium affects the mitochondrial respiratory chain and the amount of adenosine triphosphate (ATP) needed for the biosynthesis of SAM via the Yang cycle [57,58,59]. Cu^2+^ ions can cause oxidative stress, leading to changes in the methylation patterns of DNA, especially cytosines [60]. Moreover, modified bases are subject to repair. If the repair system does not work correctly, modified cytosines act as a source of DNA mutation. We also suspect that Cu^2+^ and Ag^+^ ions, as well as DNA methylation changes, may be responsible for green plant regeneration, and the tissue culture duration controls DNA methylation changes, SV and green plant regeneration. Such relationships could be predicted using the structural equation modeling (SEM) approach [61], which is widely exploited in psychology [62] but relatively rarely in biology [63] and agriculture [64,65]. The primary goal of SEM is to explain the observed variability in the data set, described by the covariance matrix, with smallest number of parameters of the postulated model for the analyzed process or phenomenon. If the tested model is confirmed using empirical data, then it is possible to estimate the strength of the relationship between the variables included in this model. These relationship strengths accurately represent the cause and effect of the process being studied. SEM, unlike other methods of statistical modeling, allows the inclusion of all interactions and interdependencies that accompany a given process or phenomenon [64]. Utilizing SEM for studying relationships between multiple factors putatively involved in the control of tissue culture-induced variation (TCIV), components of biochemical pathways affected by in vitro anther culture and green plant regeneration is a sophisticated combination of methods allowing better understanding of complex relationships in anther culture. Such knowledge may have practical implications, as it may help to gain control over the number of green plants regenerated via in vitro anther culture and the level of DNA SV in these plants.

In this study, we evaluated the relationships between different factors affecting SV and green plant regeneration via anther culture in barley using the SEM approach.

## 2. Materials and Methods

Data used for current analysis and model generation were based on plant materials evaluation, DNA isolation and metAFLP and DArTSeqMet analysis, as well as the FTIR spectroscopy conducted and described earlier [48,50,56].

Variables used for SEM, their descriptive statistics are given in Table 1, whereas Pearson’s linear correlation coefficients in Table 2 (see Results).

To eliminate constants from the structural equation model, the difference between observation and mean value for variables was used instead of raw data.

The structural equation model was implemented in IBM SPSS^®^ Amos™ 20 [66] computer software. The maximum likelihood (ML) estimation with the Levenberg–Marquardt iteration method was used to optimize the parameters of postulated models [67,68,69].

## 3. Results

Molecular and phenotypic data as well as the results of mediation analysis presented in our earlier study [50] were used here to create a generalized model predicting essential relationships between selected genetic and biochemical features and Cu^2+^ and Ag^+^ ions concentrations in barley (*Hordeum vulgare* L.) anther cultures.

### 3.1. Characterization of Input Data

Construction of the structural equation model was based on nine variables evaluated employing metAFLP characteristics, DArTseqMet marker-based methylation changes, ATR-FTIR spectroscopy, Cu^2+^ and Ag^+^ ion concentrations and Time. The descriptive statistics of the analyzed variables, including mean, variance, skewness and kurtosis are presented in Table 1.

All of the analyzed variables were quantitative and met the conditions set out and the Lindeberg–Lévy theorem [70]. It can, therefore, be assumed that the distribution of these variables is asymptotically convergent with the theoretical normal distribution. Among these variables, CHG_DMV and SV showed relatively high skewness and kurtosis in the analyzed random sample.

Kenny [71] indicated that variables with non-normal distribution, especially those with high kurtosis, inflate the chi-square and absolute measures of fit values [72]. Moreover, the relatively small sample size (*n* = 35) was disadvantageous, as it may falsely result in non-significant chi-square statistics [73]. Therefore, the chi-square test was used only as an information criterion [74] and was not used to determine the correctness of the model. Instead, the correctness of the model should be evaluated using numerous model fit measures.

A wide range of fit measures can be used to assess the goodness of fit of a model. To interpret the fitness of the model, all possible limitations resulting from the specificity of the data and the model itself were taken into account. For example, [73] showed that some goodness-to-fit indices are relatively stable with small sample sizes, whereas others such as root mean square error of approximation (RMSEA) and standardized root mean square residual (SRMR), increase with smaller sample sizes. Additionally, [71] suggested that small sample size can be used for simple models and models without latent variables. Parsimonious fit measures such as parsimonious normed fit index (PNFI) and parsimonious comparative fit index (PCFI) include in their construction an element of model complexity. These measures are used when comparing models with different degrees of freedom (df). The higher the value of these indices, the better the model [75,76].

SEM allows creating a statistical description of complex causal relationships. Compared with other commonly used methods (such as regression analysis and Write’s path analysis), SEM permits the inclusion of more complex relationships between variables, including those with exogenous variables, in the model. The correlation coefficient analysis allows the evaluation of the occurrence and complexity of the relationship within the studied process or phenomenon. Pearson linear correlation coefficients (Table 2) revealed correlations between the analyzed traits, indicating a complex relationship within the analyzed data.

#### 3.1.1. Model Specification and Estimation

**Figure 1:**
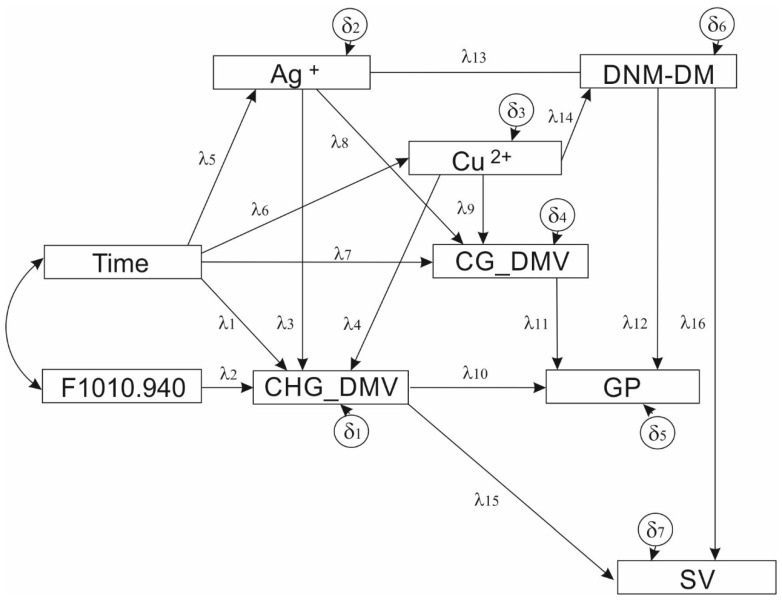
The path diagram is a graphical representation of the structural equation model. Rectangles represent observable endo- and exogenous variables, whereas circles are random errors for endogenous variables (denoted by delta symbols). One-way arrows illustrate causal relationships between variables. Path coefficients are marked by lambda parameters. The semicircular bidirectional arrow represents the presence of covariance between exogenous variables in the model. Time represents the duration of anther culture; CHG_DMV and CG_DMV represent the DNA demethylation of the CHG and CG sequence contexts, respectively, determined using metAFLP markers [9,48]; SV represents sequence variation evaluated using the metAFLP approach; DNM-DM is the difference between de novo methylation and DNA demethylation, evaluated using the DArTseqMet approach [54,56]; GP is the number of green plants regenerated per 100 anthers via in vitro anther culture of barley [43]; Cu^2+^ and Ag^+^ represent ion concentrations present in the tissue culture medium [9,43]. The F1010.940 is the ATR-FTIR spectral range assigned to β-glucans (1010–940 cm^−1^) [50].

SEM revealed relationships between all variables included in Table 1. The postulated model contained two exogenous variables (F1010.940 and Time), seven endogenous variables (Cu^2+^, Ag^+^, CHG_DMV, CG_DMV, DNM-DM, SV and GP) with seven random errors, one covariance effect and 16 non-recursive relations (Figure 1).

#### 3.1.2. Model Description

The structural equation model (SEM) illustrates (Figure 1) the role of copper and silver ions added to the in vitro induction medium during barley plant regeneration via anther culture Copper ions influence both SV and GP. Based on the model, copper ions affect CHG_DMV. The CHG_DMV directly affects SV and GP. Time of anther culture also plays an essential role in the action of the ion. Silver ions directly influence CHG_DMV, and the DNM-DM is involved in GPs. Moreover, DNM-DM may also influence SV. The action of silver ions is controlled by Time of anther culture. The F1010.940 is to be linked to the CHG_DMV.

#### 3.1.3. Model Matching

The quality of the structural equation model is assessed by analyzing the fit of the relationship system described by the postulated model to the mutual relationship system within the data derived from the real assessment of a given process or phenomenon. Various fit statistics are used for this purpose.

The tested model met all the convergence criteria. The fit statistics (Table 3) were proper. The chi-square statistic indicated that the postulated model satisfactorily confirmed the empirical data.

The SRMR value was relatively low but failed to meet the criterion defined by Hu and Bentler [77,78]. In multiple regression analysis, the goodness-of-fit index (GFI) and adjusted goodness-of-fit index (AGFI) can be interpreted analogously to the coefficient of determination [79]. Values of both AGFI and GFI were high, and GFI was close to the lowest limit (0.9), implying that the postulated model describes approximately 90% of the variability observed in the dataset. The normed fit index (NFI) and relative fit index (RFI) did not exceed the limit reported in the literature (0.95) [77,78]. The same was true for the incremental fit index (IFI), non-normed fit index (NNFI) and comparative fit index (CFI), but not for NNFI. However, applying the rule described by MacCallum and colleagues [80], obtained RMSEA results testified that the postulated model showed a good fit.

#### 3.1.4. Estimation of Model Parameters

After confirming that the postulated model is correct (i.e., it describes the system of dependencies within empirical data), the estimated values of the structural equation model parameters were used for a detailed description of the type and nature of causal relationships.

The values of individual path coefficients were estimated. Some of these coefficients were not statistically significant; however, their removal resulted in a substantial reduction in the quality of matching between the postulated model and the empirical data. Therefore, we decided to retain all the paths in the model (Table 4).

Absolute values of the standardized path coefficients explaining the relationships between various variables were calculated. The highest value was obtained for the relationship between CHG_DMV and SV, followed by that between Ag^+^ and CHG_DMV, Cu^2+^ and DNM_DM, Time and CG_DMV, CG_DMV and GP, Cu^2+^ and CHG_DMV and lastly Ag^+^ and CG_DMV.

No significant covariance was found between exogenous variables. The estimated variances for random components (δ1–δ7) and variances of exogenous variables were significantly different from zero.

The significance of model parameters expressing direct, indirect and total effects on standardized coefficients is summarized in Table 5. The CHG_DMV variable showed the highest dependence on Ag^+^ (*β* = −0.695) and Cu^2+^ (*β* = −0.481) ions, and these were direct effects. The SV variable showed the greatest dependence on CHG_DMV (*β* = −0.985), an immediate (direct) effect, followed by Ag^+^ (*β* = 0.644) and Cu^2+^ (*β* = 0.356) ions, which represented indirect effects. The GP variable showed the highest dependence on CG_DMV (*β* = −0.563; direct effect), followed by Ag^+^ ions (*β* = 0.245; indirect effect).

The relationship of the CG_DMV variable with Time (*β* = 0.591) and Ag^+^ ion concentration (*β* = −0.402), which are both direct effects, showed the greatest weight. The DNM_DM variable depended the most on Cu^2+^ (*β* = 0.646) and Ag^+^ (*β* = 0.220) ion concentrations (direct effects).

## 4. Discussion

The addition of Cu^2+^ and Ag^+^ ions to the induction and regeneration medium influences plant regeneration via anther culture. The process of tissue culture is predisposed to SV [48] because of changes in DNA methylation patterns [56]. Cu^2+^ and Ag^+^ ions affect the mitochondrial complex IV [81,82] and the functioning of the Yang cycle [58] (and consequently SAM production). Cu^2+^ ions also induce mutations in CG and CHG sequence contexts [50,56] because of oxidative stress-triggered modification of 5 mC [60]. However, tissue culture-induced SV probably starts much earlier. The presence of mannitol in the culture medium causes starvation stress. Under this condition, β-glucans, which are present between the cell wall [50] and cell membrane of embryogenic microspores [22], are probably utilized as a source of glucose [48] for the production of acetyl-coenzyme A, which is required for the Krebs cycle [83]. Additionally, DNA methylation pattern changes induced during the gametophytic to sporophytic switch [5,84], as well as Cu^2+^ and Ag^+^ ion concentrations in the culture medium, determine the number of green plants regenerated via anther culture. Finally, our model also assumed that Time influences SV and GP. Although the link between tissue culture-induced SV and green plant regeneration was not yet established, some elements of such a model had been published previously [48,50,56], which were linked using the SEM approach in this study.

According to the structural equation model, β-glucans (F1010.940 FTIR spectral range) and CHG_DNMV negatively influenced CHG_DMV and SV, respectively. We failed to include the F710.690 FTIR spectral range (preliminarily assigned to SAM) into the structural equation model. This could be explained by the relatively small sample size and low cellular SAM concentration (<100 mM), which is technically challenging to measure [85]. Fluctuations in SAM concentration may not be sufficient to be significant in a model including many variables under small sample size conditions. However, under starvation conditions, the concentration of SAM increases because of the universal energy-sensing regulator Snf1, which is the yeast (*Saccharomyces cerevisiae*) ortholog of AMP-activated protein kinase (AMPK) [86].

Effects of Cu^2+^ and Ag^+^ ion concentrations on CHG_DMV and Time were less pronounced than those of β-glucans. Thus, demethylation of the CHG context is not under the robust control of metal ions but is influenced by β-glucans and SV. The model also assumes that Time influences CG_DMV via the action of Cu^2+^ ions. However, the effects describing this relation are not very strong. The effect of Ag^+^ ions, (acting as a mediator), on Time and CG_DMV was much more pronounced than that of Cu^2+^ ions. This is in agreement with the fact that Ag^+^ ions may replace Cu^2+^ ions, for example, in the mitochondrial complex IV, which affects the methionine cycle. Interestingly, in contrast to our results of mediation analysis [50], changes in CG_DMV did not contribute to SV.

All of the relationships that contributed to SV were based on metAFLP characteristics and did not seem to be linked to GP. However, by using DArTseqMet markers, we showed that the difference between de novo methylation and DNA demethylation (DNM-DM), influences SV. Moreover, the DNM-DM variable was affected much more by Cu^2+^ ions than by Ag^+^ ions. The model predicts that the number of green plants is under the limited control of the DNM-DM variable. Thus, the model presented in this study can explain the SV and is congruent with the role of DNA methylation in SV. We assumed that CHG_DMV rather than CG_DMV participates in SV. However, further investigation is needed to understand whether SV is mainly caused by point mutations or transposable elements. It should be stressed that CHG_DMV only slightly affected GP. This suggests that green plant regeneration is relatively independent of changes in DNA methylation patterns due to anther culture.

It must be emphasized that the model presented in this study might not be able to identify all the relationships affecting SV and GP, possibly because of the limitations of FTIR spectroscopy, non-normal distribution of some variables and/or small sample size. Additional factors need to be incorporated into the model to link these phenomena.

It is always important to question whether the results of SEM analysis are reliable. Based on the analysis of the obtained goodness-of-fit coefficients (Table 4), we conclude that the postulated model is correct and adequately describes the complex relationships between the analyzed variables. The presented model indicates that by manipulating Cu^2+^ and Ag^+^ ion concentrations, we can predict the biochemical factors that induce SV and to some extent increase the GP. We also demonstrated that the callose layer is a crucial participant in the model under varying Cu^2+^ and Ag^+^ concentrations. It would be of value to test whether there is a correlation between the amount of callose present in embryogenic microspore culture and the number of regenerated plants. If so, the presence of callose could be used as an indicator of the capacity of microspores to switch from the gametophytic to sporophytic fate. Callose could also be used to identify genotypes suitable for anther-dependent plant regeneration. Thus, understanding the relationships that influence sequence (or total tissue culture-induced) variation and GP is of practical value to plant breeders and scientists working on tissue culture.

Possibly the most important outcome of the structural equation model is the opportunity to predict the level of outcomes (start and end of the path, see Table 5) if the model parameters (independent variables: start of the path) are changed, thus analyzing direct effects. For example, in this study, an increase in F1010.940 by one unit caused a reduction in CHG_DMV by 6.23 units, whereas a reduction in CHG_DMV decreased the SV by 9.8 units. This implies that Cu^2+^ ions have a limited influence on CHG_DMV, as increasing the Cu^2+^ ion concentration by one unit decreased CHG_DMV by 0.037 units. Thus, the model predicts that when β-glucans act as the source of carbon, the larger the subintinal callose layer (under starvation conditions), the lower the CHG_DMV and SV. Furthermore, the role of Time is also limited, as an increase in its value by one unit increased CHG_DMV only by 0.014 units.

Detailed analysis of estimates presented in Table 5 enables the analysis of the paths of influence of individual variables. For example, Time directly influenced Ag^+^ ions (*b* = 0.800), implying that an increase in Time by one unit acts as though the Ag^+^ ion concentration increased by 0.8 units); similarly, an increase in Time by one unit acts as if Cu^2+^ ion concentration decreased by 0.023 units, and increased CHG_DMV and CG_DMV by 0.014 and 0.017 units, respectively. Additionally, Time influenced CHG_DMV (*b* = −0.005) indirectly through Ag^+^ and Cu^2+^ ions, GP (*b* = −0.042) via CHG_DMV and CG_DMV, and SV (*b* = −0.088) via CHG_DMV. Similarly, the Ag^+^ ions directly influenced CHG_DMV (*b* = 0.007) and CG_DMV (*b* = −0.003), but indirectly influenced GP (*b* = 0.008) through CHG_DMV and CG_DMV and similarly SV (*b* = 0.067) via CHG_DMV. Cu^2+^ ions were characterized by similar paths of dependence. Cu^2+^ ions directly affected CHG_DMV (*b* = −0.037) and CG_DMV (*b* = −0.009) but indirectly influenced GP (*b* = 0.042) and SV (*b* = 0.275) via CHG_DMV and CG_DMV, respectively. Investigating the individual paths of dependence allows us to characterize the changes taking place in the causal model described, if the values of causal variables, such as Time, Ag^+^ or Cu^2+^ show any change.

While SV was evaluated using metAFLP markers [48,50], GP was examined using MSAP markers [56]. The two marker systems are based on distinct marker platforms utilizing different endonucleases. The metAFLP is based on *Kpn*I and *Acc*65I isoschizomers, whereas the MSAP approach and the variant involving the DArTseqMet approach are based on *Hpa*II and *Msp*I endonucleases. Additionally, the metAFLP and DArTseqMet methods recognize distinct DNA methylation patterns; while metAFLP can distinguish between methylation changes affecting CHH, CHG and CG contexts, DArTseqMet can capture only CHG and CG alterations. Moreover, marker systems recognize different restriction sites. Genetic mapping in cereals demonstrated that many AFLP markers based on the *Eco*RI endonuclease mapped to genomic regions other than those generated using *Pst*I [87]. Although undocumented, we speculate that *Kpn*I–*Acc*65I and *Hpa*II–*Msp*I endonuclease pairs will generate markers with non-random distribution along the chromosomes, thus potentially mapping to different genomic regions. Thus, the two marker systems may identify distinct phenomena, similar to the models depicting SV [48,50] and green plant regeneration [56]. Implementation of distinct marker systems in combination with FTIR spectroscopy will be highly valuable for evaluating relationships between multiple factors affecting tissue culture and for predicting the roles of these factors in SV and green plant regeneration.

## 5. Conclusions

We evaluated the structural equation model describing complex relationships between different factors including DNA methylation changes, SV, β-glucans, Cu^2+^ and Ag^+^ ions, Time and GP. The model was constructed on theoretical bases concerning DNA methylation changes, sources of DNA mutations, the effect of Cu^2+^ and Ag^+^ ion concentrations on DNA methylation and GP. The theoretical background was also supported by our moderation and mediation analysis, partly linking the variables. Although the structural equation model was evaluated based on a relatively small sample size (because of experimental limitations), analysis of the model using a wide range of measures of fit suggests that the model is reliable. Nevertheless, the use of this model on larger sample sizes and different cereal species is required to verify its general application in anther culture-derived regenerants. The presented model predicts the outcome of a change in tissue culture conditions.

## Figures and Tables

**Table 1:** Descriptive statistics of the analyzed variables and their presence in postulated models (calculated based on data in Table 1).

Variable	Descriptive Statistics			
	Mean	Variance	Skewness	Kurtosis
[F1010.940] ^1^	0.035	0.000	0.531	−0.689
[Cu^2+^]	4.751	17.205	0.123	−1.573
[Ag^+^]	20.286	667.546	0.900	−1.129
[DNM-DM]	0.794	6.089	−0.804	−0.048
[CG_DMV]	0.391	0.056	0.170	0.219
[CHG_DMV]	0.940	0.098	−2.393	5.433
[SV]	3.952	9.585	2.849	7.049
[GP]	1.123	0.715	0.835	−0.424
[Time]	27.800	35.988	0.057	−1.657

^1^ F1010.940: the area integrated absorbance for spectral ranges between 1010 and 940 cm^−1^ evaluated by attenuated total reflection Fourier transform infrared (ATR-FTIR) spectroscopy. Cu^2+^ and Ag^+^ are copper and silver ion concentrations; DNM, de novo methylation; DM: demethylation; DNM-DM: changes in methylation; CG_DMV and CHG_DMV: demethylation of the CG and CHG contexts; SV: sequence variation; GP: number of green plants regenerated per 100 anthers. Square brackets are given to indicate that the respective parameters are variables in SEM.

**Table 2:** Pearson’s linear correlation coefficients for analyzed variables.

Variable	[F1010.940] ^1^	[Cu^2+^]	[Ag^+^]	[DNM_DM]	[CG_DMV]	[CHG_DMV]	[SV]	[GP]	[T]
[F1010.940]	1.000								
[Cu^2+^]	0.338 a *	1.000							
[Ag^+^]	−0.017	−0.107	1.000						
[DNM_DM]	−0.128	0.476 **	0.166	1.000					
[CG_DMV]	−0.157	−0.055	−0.208	−0.023	1.000				
[CHG_DMV]	−0.405 *	−0.438**	−0.508 **	−0.231	0.590 **	1.000			
[SV]	0.472 **	0.418 *	0.478 **	0.080	−0.391 *	−0.887 **	1.000		
[GP]	0.050	0.347 *	0.157	0.210	−0.315	−0.251	0.240	1.000	
[Time]	−0.079	−0.002	0.160	0.114	0.593 **	0.306	−0.252	−0.138	1.000

^1^ Asterisks indicate significant differences (* *p* < 0.05; ** *p* < 0.01).

**Table 3:** Summary of the analyzed structural equation model.

Parameter	Postulated Model
Degrees of freedom (df)	19
Chi-square	21.125
*p*-value	0.330
Root Mean Squares Residuals (RMR)	2.836
Standardized Root Mean Squares Residuals (SRMR)	0.134
Goodness-of-Fit Index (GFI)	0.862
Adjusted Goodness-of-Fit Index (AGFI)	0.673
Normed Fit Index (NFI)	0.647
Relative Fit Index (RFI)	0.331
Incremental Fit Index (IFI)	0.948
Non-Normed Fit Index (NNFI)	0.831
Comparative Fit Index (CFI)	0.911
Parsimonious Normed Fit Index (PNFI)	0.341
Parsimonious Comparative Fit Index (PCFI)	0.481
Root Mean Square Error of Approximation (RMSEA)	0.057

**Table 4:** Path coefficients, variances and covariances for the analyzed model.

Parameter	Effect			Estimate (*b*)	Standard Error	Test Statistic	Standardized Estimate (*β*)
*Path coefficients*							
*λ*_1_	[Time]	→	[CHG_DMV]	0.014	0.005	2.641 **	0.284
*λ*_2_	[F1010.940]	→	[CHG_DMV]	−6.230	5.127	−1.215	−0.139
*λ*_3_	[Ag^+^]	→	[CHG_DMV]	−0.007	0.001	−6.508 **	−0.695
*λ*_4_	[Cu^2+^]	→	[CHG_DMV]	−0.037	0.009	−4.180 **	−0.481
*λ*_5_	[Time]	→	[Ag^+^]	0.800	0.834	0.959	0.173
*λ*_6_	[Time]	→	[Cu^2+^]	−0.023	0.127	−0.179	−0.036
*λ*_7_	[Time]	→	[CG_DMV]	0.018	0.005	3.555 **	0.592
*λ*_8_	[Ag^+^]	→	[CG_DMV]	−0.003	0.001	−2.404 *	−0.402
*λ*_9_	[Cu^2+^]	→	[CG_DMV]	−0.009	0.009	−1.011	−0.179
*λ*_10_	[CHG_DMV]	→	[GP]	0.055	0.676	0.082	0.019
*λ*_11_	[CG_DMV]	→	[GP]	−2.596	0.953	−2.723 **	−0.563
*λ*_12_	[DNM_DM]	→	[GP]	0.046	0.068	0.684	0.141
*λ*_13_	[Ag^+^]	→	[DNM-DM]	0.021	0.013	1.586	0.220
*λ*_14_	[Cu^2+^]	→	[DNM-DM]	0.454	0.109	4.161 **	0.646
*λ*_15_	[CHG_DMV]	→	[SV]	−9.803	1.047	−9.366 **	−0.985
*λ*_16_	[DNM_DM]	→	[SV]	−0.200	0.106	−1.880	0.141
*Covariances*							
φ1	[F1010.940]	↔	[Time]	0.002	0.007	0.347	0.074
*Variances*							
*δ*_1_				0.018	0.005	3.346 **	
*δ*_2_				616.775	151.285	4.077 **	
*δ*_3_				11.636	3.407	3.415 **	
*δ*_4_				0.015	0.005	3.018 **	
*δ*_5_				0.396	0.124	3.199 **	
*δ*_6_				3.083	0.930	3.313 **	
*δ*_7_				1.120	0.328	3.418 **	
[Time]				29.723	7.845	3.789 **	
[F1010.940]				0.000	0.000	3.015 **	

*—significant at *p* < 0.05; **—significant at *p* < 0.01.

**Table 5:** Direct, indirect and total effects for the analyzed model.

Effect			Estimates (*b*)			Standardized Estimates (*β*)		
			Direct Effect	Indirect Effects	Total Effects	Direct Effect	Indirect Effects	Total Effects
[CHG_DMV]								
[Time]	→	[CHG_DMV]	0.014	−0.005	0.009	0.284	−0.103	0.181
[F1010.940]	→	[CHG_DMV]	−6.230	—	−6.230	−0.139	—	−0.139
[Ag^+^]	→	[CHG_DMV]	0.007	—	0.007	−0.695	—	−0.695
[Cu^2+^]	→	[CHG_DMV]	−0.037	—	−0.037	−0.481	—	−0.481
[Ag^+^]								
[Time]	→	[Ag^+^]	0.800	—	0.800	0.173	—	0.173
[Cu^2+^]								
[Time]	→	[Cu^2+^]	−0.023	—	−0.023	−0.036	—	−0.036
[SV]								
[CHG_DMV]	→	[SV]	−9.803	—	−9.803	−0.985	—	−0.985
[Time]	→	[SV]	—	−0.088	−0.088	—	−0.181	−0.181
[F1010.940]	→	[SV]	—	61.073	61.073	—	0.137	0.137
[Ag^+^]	→	[SV]	—	0.067	0.067	—	0.644	0.644
[Cu^2+^]	→	[SV]	—	0.275	0.275	—	0.356	0.356
[DNM_DM]	→	[SV]	−0.200	—	−0.200	−0.182	—	−0.182
[GP]								
[CHG_DMV]	→	[GP]	0.055	—	0.055	0.019	—	0.019
[Time]	→	[GP]	—	−0.042	−0.042	—	−0.292	−0.292
[F1010.940]	→	[GP]	—	−0.344	−0.344	—	−0.003	−0.003
[Ag^+^]	→	[GP]	—	0.008	0.008	—	0.245	0.245
[Cu^2+^]	→	[GP]	—	0.042	0.042	—	0.183	0.183
[CG_DMV]	→	[GP]	−2.596	—	−2.596	−0.563	—	−0.563
[DNM_DM]	→	[GP]	0.046	—	0.046	0.141	—	0.141
[CG_DMV]								
[Ag^+^]	→	[CG_DMV]	−0.003	—	−0.003	−0.402	—	−0.402
[Cu^2+^]	→	[CG_DMV]	−0.009	—	−0.009	−0.179	—	−0.179
[Time]	→	[CG_DMV]	0.017	—	0.017	0.591	—	0.529
[DNM_DM]								
[Ag^+^]	→	[DNM_DM]	0.021	—	0.021	0.220	—	0.220
[Cu^2+^]	→	[DNM_DM]	0.454	—	0.454	0.646	—	0.646
[Time]	→	[DNM_DM]	—	0.006	0.006	—	0.015	0.015

## Data Availability

Not applicable.

## Abbreviations

AGFI	Adjusted Goodness-of-Fit Index
AMPK	AMP-Activated Protein Kinase
ATP	Adenosine Triphosphate
ATR-FTIR	Attenuated Total Reflection Fourier Transform Infrared spectroscopy
CFI	Comparative Fit Index
DArTseqMet	Diversity Arrays Technology Sequencing Methylation Analysis
DM	Demethylation
DNM	De Novo methylation
ET	Endogenous ethylene
ETR1	Ethylene receptor1
GFI	Goodness-of-Fit Index
GP	Green Plant
IFI	Incremental Fit Index
metAFLP	Methylation-Sensitive Amplified Fragment Length Polymorphism
ML	Maximum Likelihood
MSAP	Methylation Sensitive Amplification Polymorphism
NFI	Normed Fit Index
NNFI	Non-Normed Fit Index
PCFI	Parsimonious Comparative Fit Index
PNFI	Parsimonious Normed Fit Index
RFI	Relative Fit Index
RMR	Root Mean Squares Residuals
RMSEA	Root Mean Square Error of Approximation
ROS	Reactive Oxygen Species
SAM	S-Adenosyl-L-Methionine
SEM	Structure Equatation Modeling
SRMR	Standardized Root Mean Squares Residuals
SV	Sequence Variation
TCA	Tricarboxylic Acid Cycle
TCIV	Tissue Culture-Induced Variation
